# GENETICS IN ENDOCRINOLOGY: Approaches to molecular genetic diagnosis in the management of differences/disorders of sex development (DSD): position paper of EU COST Action BM 1303 ‘DSDnet’

**DOI:** 10.1530/EJE-18-0256

**Published:** 2018-07-04

**Authors:** L Audí, S F Ahmed, N Krone, M Cools, K McElreavey, P M Holterhus, A Greenfield, A Bashamboo, O Hiort, S A Wudy, R McGowan, the EU COST Action

**Affiliations:** 1 Growth and Development Research Unit, Vall d’Hebron Research Institute (VHIR), Center for Biomedical Research on Rare Diseases (CIBERER), Instituto de Salud Carlos III, Barcelona, Spain; 2 Developmental Endocrinology Research Group, University of Glasgow, Glasgow, UK; 3 Academic Unit of Child Health, Department of Oncology and Metabolism, University of Sheffield, Sheffield Children’s Hospital, Western Bank, Sheffield, UK; 4 Department of Paediatric Endocrinology, Ghent University Hospital, Paediatrics and Internal Medicine Research Unit, Ghent University, Ghent, Belgium; 5 Human Developmental Genetics, Institut Pasteur, Paris, France; 6 Division of Pediatric Endocrinology and Diabetes, University Hospital of Schleswig-Holstein and Christian Albrechts University, Kiel, Germany; 7 Mammalian Genetics Unit, Medical Research Council, Harwell Institute, Oxfordshire, UK; 8 Division of Paediatric Endocrinology and Diabetes, Department of Paediatric and Adolescent Medicine, University of Lübeck, Lübeck, Germany; 9 Division of Pediatric Endocrinology and Diabetology, Steroid Research & Mass Spectrometry Unit, Laboratory for Translational Hormone Analytics, Center of Child and Adolescent Medicine, Justus-Liebig-University, Giessen, Germany; 10 Department of Clinical Genetics, Laboratories Building, Queen Elizabeth University Hospital, Glasgow, UK

## Abstract

The differential diagnosis of differences or disorders of sex development (DSD) belongs to the most complex fields in medicine. It requires a multidisciplinary team conducting a synoptic and complementary approach consisting of thorough clinical, hormonal and genetic workups. This position paper of EU COST (European Cooperation in Science and Technology) Action BM1303 ‘DSDnet’ was written by leading experts in the field and focuses on current best practice in genetic diagnosis in DSD patients. Ascertainment of the karyotpye defines one of the three major diagnostic DSD subclasses and is therefore the mandatory initial step. Subsequently, further analyses comprise molecular studies of monogenic DSD causes or analysis of copy number variations (CNV) or both. Panels of candidate genes provide rapid and reliable results. Whole exome and genome sequencing (WES and WGS) represent valuable methodological developments that are currently in the transition from basic science to clinical routine service in the field of DSD. However, in addition to covering known DSD candidate genes, WES and WGS help to identify novel genetic causes for DSD. Diagnostic interpretation must be performed with utmost caution and needs careful scientific validation in each DSD case.

## Introduction

Differences or disorders of sex development (DSD) arise during embryonic and foetal development. They may be caused by (1) numerical or structural variations in sex chromosomes, (2) variations in genes involved in gonadal and/or genital development (leading to inactivation or activation), (3) disorders in gonadal and/or adrenal steroidogenesis (1), (4) maternal factors (endogenous or exogenous) or (5) endocrine disruptors that can interfere with genital development (2, 3). A final, yet putative category would consist of cases resulting from epigenetic changes that are predicted to disrupt gene expression in the foetal period. Many causes are genetically determined and should be analysed when a genetic cause for a DSD is sought (4, 5, 6, 7). Reaching a molecular diagnosis is important as it may inform patient management in relation to possible gender development, assessment of adrenal and gonadal function, gonadal cancer risk, associated morbidity as well as long-term outcomes. Determining the aetiology is often useful for families, as it provides information about risk of recurrence (8, 9, 10).

This paper proposes practical recommendations for the approach to a genetic diagnosis of DSD, taking into account the clinical and biochemical phenotypes, if available, and the rapidly developing genetic technologies that continuously improve the diagnostic yield in this heterogeneous group of conditions. Diagnosis of DSD belongs to the most complex fields in medicine and requires an integrated multidisciplinary approach consisting in a synoptic view of clinical phenotype, biochemical (hormonal) constellation and molecular datasets. This position paper presents current best practice in the molecular genetic diagnosis of DSD and is the result of a truly Europe-wide concerted action of leading DSD specialists within EU COST Action BM 1303 ‘DSDnet’.

## General approach to the genetic diagnosis of DSD

If DSD is considered as all types of atypical genitalia at birth together with all discordances among chromosomal, gonadal and genital sexes, the prevalence of DSD reaches about 5 per 1000 births, with 73% of them being boys with hypospadias (11). Among children with atypical genitalia, 75% will have a 46,XY karyotype, 10–15% a 46,XX and the remainder will have structural or numerical anomalies of the sex chromosomes (12). The most frequent autosomal recessive condition associated with monogenic DSD in individuals with 46,XX karyotype is congenital adrenal hyperplasia (CAH) due to 21-hydroxylase deficiency. It accounts for approximately 90–95% of 46,XX individuals (13). Other well-characterised monogenic 46,XX DSDs are extremely rare (see list in Table 1), and their molecular diagnosis has long been challenging.

**Table 1:** Genes involved in monogenic disorders/different sex development (DSD).

Clinical diagnosis	Gene (locus)	OMIM (inheritance) (additional phenotype)
**1. 46,XX with disorders of gonadal development: gonadal dysgenesis, ovotesticular DSD, testicular DSD**		
		
Gonadal dysgenesis	*BMP15* (Xp11.22)	300510 (D)
Testicular DSD	*FGF9* (13q12.11)	600921 (AD:dup) (single case description)
Gonadal dysgenesis	*FOXL2* (3q22.3)	608996 (AD) (blepharophimosis, epicanthus inversus and ptosis, types I and II)
Testicular DSD	*NR2F2* (15q26.2)	615779 (AD) (congenital heart defects, congenital diaphragmatic hernia, blepharo-phimosis-ptosis-epicanthus inversus syndrome)
(1) Gonadal dysgenesis	(1) *NR5A1* (9q33.3)	612964 (AD)
(2) Ovotesticular DSD	(2) *NR5A1* (9q33.3) (p.Arg92Trp)	617480 (AD)
(3) Testicular DSD	(3) *NR5A1* (p.Arg92Trp)	
Gonadal dysgenesis	*NUP107* (12q15)	607617 (AR) (described in consanguineous family; other phenotypes with nephrotic syndrome)
Ovotesticular DSD	*RSPO1* (1p34.3)	610644 (AR) (palmoplantar hyperkeratosis, squamous cell carcinoma of skin)
(1) Ovotesticular DSD	*SOX3* (Xq27.1)	313430 (XL:dup)
(2) Testicular DSD		
(1) Ovotesticular DSD	*SOX9* (17q24.3)	278850 (AD:dup)
(2) Testicular DSD		
Ovotesticular DSD or testicular DSD	*SOX10* (22q13.1)	609136 (AD:dup) (Waardenberg and Hirschsprung syndromes, peripheral neuropathy)
(1) Ovotesticular DSD	*SRY* (Yp11.2)	400045 (T)
(2) Testicular DSD		
(1) Ovotesticular DSD	*WNT4* (1p36.12)	158330 (AD)
(2) Testicular DSD		611812 (AR): SERKAL (sex reversal dysgenesis of kidneys, adrenals and lung) syndrome, lethal when biallelic
2. 46,XY with disorders of gonadal development: gonadal dysgenesis (GD), complete or partial (C/P-GD)		
P-GD	*ARX* (Xp21.3)	300215 (XL:D) (Lissencephaly, epilepsy, intellectual deficiency)
P-GD	*ATRX* (Xq21.1)	300032 (D:del) (intellectual deficiency, α-thalassemia)
Ovaries or C-GD	*CBX2* (17q25.3)	613080 (AR)
C-GD or P-GD	*DAX1* (*NR0B1*) (Xp.21)	300018 (XL:dup)
C-GD or P-GD	*DHH* (12q13.12)	233420/607080 (AR/AD) (minifascicular neuropathy)
C-GD or P-GD	*DMRT1* (9p24.3)	602424 (AD:del) (with or without intellectual deficiency)
P-GD	*EMX2* (10q26.11)	600035 (AD:del) (intellectual deficiency, kidney agenesis)
C-GD or P-GD	*ESR2* (14q23.2–q23.3)	601663 (biallelic and monoallelic)
C-GD or P-GD	*FGFR2* (10q26.13)	176943 (AD) (cranyosinostosis)
C-GD or P-GD	*GATA4* (8p23.1)	615542 (AD) (with or without congenital heart disease)
C-GD	*HHAT* (1q32.2)	605743 (AR) (single familial case description) (short stature, generalised chondrodysplasia, muscle hypertophy, myopia, intellectual deficiency)
C-GD or P-GD	*MAP3K1* (*MEKK1*) (5q11.2)	613762 (AD)
C-GD or P-GD	*NR5A1* (9q33.3)	612965 (AD)/(AR) (rarely primary adrenal failure, hypogonadotropic hypogonadism)
C-GD or P-GD	*SOX9* (17q24.3)	114290 (AD) (campomelic dysplasia)
C-GD or P-GD	*SRY* (Yp11.2)	400044 (D)
C-GD or P-GD	*TSPYL1* (6q22.1)	608800 (AR) (sudden infant death with dysgenesis of the testes, SIDDT syndrome)
Ovaries or ovotesticular DSD or C-GD	*WNT4* (1p36.12)	603490 (AD:dup)
		607102 (AD)
P-GD	*WT1* (11p.13)	(1) 194072 (del 11.p13: WAGR syndrome)(2) 194080 (inactivation: Denys-Drash syndrome)(3) 136680 (splicing: Frasier syndrome)
C-GD or P-GD or ovotesticular DSD	*ZFPM2* (*FOG2*) (8q23.1)	616067 (AD) (with or without congenital heart disease)
C-GD or P-GD	*ZNRF3* (22q12.1)	612062 (AD)
3. 46,XX DSD with androgen excess		
		
CAH with 3β-hydroxysteroid dehydrogenase type 2 deficiency	*HSD3B2* (1p12)	201810 (AR) (adrenal and gonadal deficiency)
CAH with 21-hydroxylase deficiency	*CYP21A2* (6p21.33)	201910 (AR) (adrenal deficiency)
CAH with 11β-hydroxylase deficiency	*CYP11B1* (8q24.3)	202010 (AR) (adrenal deficiency)
P450-oxidoreductase deficiency	*POR* (7q11.23)	201750 (AR) (antley-Bixler syndrome, craniosynostosis ±)
Aromatase deficiency	*CYP19A1* (15q21.2)	613546 (AR) (maternal and foetal virilisation)
Oestrogen insensitivity	*ESR1* (6q25.1–q25.2)	615363 (AR) (overgrowth, osteoporosis, polycystic ovary syndrome)
Glucocorticoid insensitivity	*GRα* (*NR3C1*) 5q31.3	615962 (AD) (hypertension)
4. 46,XY DSD with abnormal androgen synthesis or action or isolated hypospadias or cryptorchidism		
		
Abnormal LH	*LHB* (19q13.33)	228300 (AR) (bioinactive LH)
LH/CG insensitivity	*LHCGR* (2p16.3)	238320 (AR) (Leydig cell aplasia, hypoplasia)
7-Dehydro-cholesterol desmolase deficiency	*DHCR7* (11q13.4)	270400 (AR) (Smith-Lemli-Opitz syndrome)
STAR deficiency (Lipoid CAH)	*STAR* (8p11.23)	201710 (AR)
(1) Classical form		(1) Adrenal and gonadal deficiency
(2) Non-classical form		(2) Adrenal deficiency
CAH with cholesterol desmolase deficiency	*CYP11A1* (15q24.1)	613743 (AR) (adrenal and gonadal deficiency)
CAH with 3β-hydroxysteroid dehydrogenase type 2 deficiency	*HSD3B2* (1p12)	201810 (AR) (adrenal and gonadal deficiency)
	*CYP17A1* (10q24.32)	202110 (AR)
(1) CAH with combined 17 hydroxylase/17,20-lyase deficiency		(1) CAH + hypertension + gonadal deficiency
(2) Isolated 17,20-lyase deficiency		(2) Gonadal deficiency
P450-oxidoreductase deficiency	*POR* (7q11.23)	201750 (AR) (Antley-Bixler syndrome)
Cytochrome b5 deficiency	*CYB5A* (18q22.3)	250790 (AR) (methemoglobinemia type IV)
17β-Hydroxysteroid-dehydrogenase type 3 (17-keto-reductase) deficiency	*HSD17B3* (9q22.32)	264300 (AR) (Gonadal deficiency)
5α-Reductase type 2 deficiency	*SRD5A2* (2p23.1)	264600 (AR)
Backdoor steroidogenesis deficiency	*AKR1C2* (10p15.1)	614279 (AR)
	*AKR1C4* (10p15.1)	DSD with DHT deficiency and apparent 17,20-lyase deficiency + normal *CYP17A1* and *SRD5A2*
Androgen insensitivity: Complete (CAIS) Partial (PAIS)	*AR* (Xq12)	300068/312300/300633 (XL)
X-linked hypospadias	*MAMLD1* (*CXOrf6*) (Xq28)	300758 (XL) (hypospadias)
Isolated hypospadias	*ATF3* (1q32.3)	603148 (AD ??)
Cryptorchidism	*INSL3* (19p13.11)	219050 (AD)
Cryptorchidism	*RXFP2* (*LGR8/GREAT/GPR106*) (13q13.1)	606655 (AD ??)
5. 46,XY DSD with abnormal anti-Müllerian hormone secretion or action		
		
Persistent Müllerian duct syndrome type I	*AMH* (19p13.3)	261550 (AR)
Persistent Müllerian duct syndrome type II	*AMHR2* (12q13.13)	261550 (AR)
6. 46,XX DSD with Müllerian duct abnormalities		
		
MURCS (Müllerian Aplasia, Renal aplasia, Cervico-thoracic somite abnormalities) syndrome	multigenic	601076
MRKH (Mayer-Rokitansky-Küster-Hauser) syndrome, types I and II	CNV at 17q12, 1q21.1, 22q11.21, Xq21.31	277000
Müllerian Aplasia and hyperandrogenism	*WNT4* (1p36.12)	158330 (AD)
Hand-foot-uterus syndrome	*HOXA13* (7p15.2)	140000 (AD)

??, unknown; AD, autosomal dominant; AR, autosomal recessive; CAH, congenital adrenal hyperplasia; C-GD, complete gonadal dysgenesis; CNV, copy number variation; D, dominant; Del, deletion; DSD, different sex development; Dup, duplication; P-GD, partial gonadal dysgenesis; T, translocation; XL, X-linked.

Data collected between 1966 and 2014 showed that the prevalence of females with a 46,XY karyotype is 6.4/100 000 among live-born females, with 4.1/100 000 associated with androgen insensitivity (AIS) and 1.5/100 000 having a form of gonadal dysgenesis (14). Even if clinical and biochemical investigations may adequately orient the molecular diagnosis, success in reaching a molecular diagnosis in 46,XY DSD is relatively low (5). This is most likely due to the heterogeneous clinical and hormonal presentation of many DSDs, the large number of known DSD-related genes (Table 1) with often low genotype–phenotype correlation and the increasing evidence that a significant proportion of the underlying pathogenesis may be multifactorial (15, 16). Newer molecular genetic diagnostic strategies employing high-throughput sequencing (HTS) screen large panels of known DSD-related genes simultaneously, thereby improving diagnostic yield. Further investigation by whole exome (WES) (17) or genome (WGS) sequencing may identify novel genes involved in the development of the phenotype. A comprehensive list of genes known to be involved in human 46,XY and 46,XX DSD (including gonadal dysgenesis, primary and secondary gonadal insufficiency, disordered steroidogenesis, androgen resistance, isolated urogenital anomalies and syndromic conditions associated with ambiguous genitalia) is reported along with their chromosomal locations: it amounts to 62 genes in 46,XY and 61 in 46,XX (9). However, with further advance in knowledge about embryonic and foetal development, it is likely that more candidate genes for adrenal and reproductive disorders will be identified in the near future (18).

**Figure 1:**
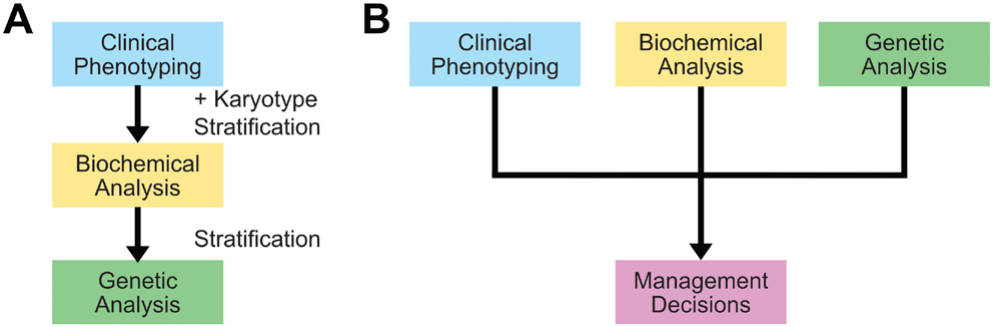
Diagnostic approaches to differences/disorders of sex development (DSD). (A) The traditional pathway approaches the diagnosis in a stepwise stratification. In particular, targeted genetic test are often only performed after biochemical guidance. (B) The recommended multidisciplinary approach in which the information on clinical phenotyping is considered in parallel with the biochemical (hormonal) data and genetic results (the karyotype and the candidate gene results) in an integrative manner.

A diagnosis of DSD may be requested prenatally, in a neonate, infant or in a prepubertal child or adolescent/adult person either because there is a family history of DSD or because the phenotype suggests the presence of DSD. Recommendations regarding clinical care are essential components of an individualised care plan and comprise: informed consent, diagnostic investigations, information and psychological support to patients and parents, transition, multidisciplinary care in adulthood, data collection across ages for the assessment of genital status, urological and gynaecological follow-up, somatic assessment, assessment of psychological outcomes and quality of life (19). Also, steroid hormone diagnostics (analytical methods and matrices, harmonisation of laboratory tests and steroid analysis in conditions associated with DSD) are essential part of DSD evaluation (20). Such recommendations have already been published as Consensus Statements by COST Action BM1303 working groups. The multidisciplinary team (paediatric or adult endocrinology, urology or gynaecology, clinical biochemistry, genetics, radiology, occasionally pathology) should be able to provide knowledge and technologies that could help in clarifying the possible genetic causes (Fig. 1). For this, the chromosomal sex should be determined at the outset because this is the basis for assignment of a DSD case to one of the three major DSD subclasses according to established DSD classification (19). Depending on this, the clinical phenotype, the hormonal constellation, the family history of DSD and the presence or absence of consanguinity, a molecular diagnosis will be sought by using the most adequate molecular testing.

### Chromosomal sex

Besides careful clinical evaluation of the patient with DSD, first-line investigation of an individual with DSD involves confirming the chromosomal sex using quantitative fluorescence polymerase chain reaction (QFPCR) and karyotype. QFPCR detects a series of markers on the sex chromosomes and has a turn-around time of 1–2 days. This technique has largely replaced fluorescence *in situ* hybridisation. The analysis of karyotype is a cytogenetic technique (involving G-banding) and is essential in the initial classification of any DSD into one of three categories: sex chromosome DSD, 46,XX DSD or 46,XY DSD. In some centres karyotyping has been replaced by array-comparative genomic hybridisation (aCGH) or SNP array, with much faster turn-around times than G-banding of 5–10 days. Unlike karyotype these techniques will not detect structural chromosomal rearrangements and may be less effective at detecting sex chromosome mosaicism. If sex chromosome DSD is identified no further genetic analysis is required. Details on different types of sex chromosomal DSDs and testing methods have been published (9).

A DSD may be suspected in the prenatal period based on family history or on the foetal genital ultrasound (US) appearance that may be discordant with the foetal genetic sex. An urgent genetic diagnosis is required if the parents consider pregnancy interruption (depending on the country’s legislation, the aetiology and the weeks of gestation). The first approach is to determine the foetal genetic sex. Non-invasive prenatal diagnosis (NIPD) can identify Y chromosome-specific markers (one within the *SRY* gene) using QFPCR in maternal peripheral blood cell-free foetal DNA from the 6th week (21), but most diagnostic laboratories recommend maternal blood sample collection from 7 weeks. If this technique is unavailable, the presence of *SRY*, the karyotype and the carrier status of a known genetically determined DSD condition can be investigated on a chorionic villus biopsy (from 9th to 11th weeks) or in amniotic fluid cells (from 15 to 20 weeks). Nowadays, the preferred approach is by NIPD and should be sought in collaboration with centres offering this test as this is not associated with risk of termination of pregnancy and as it can be performed at earlier gestational ages.

### Copy number variation analysis

Higher resolution chromosome analysis through aCGH or SNP array allows the detection of microduplications or microdeletions below the threshold of a standard karyotype (<5 Mb). In some centres, aCGH has replaced the traditional karyotype but in others aCGH or SNP array is used as part of second-line investigations for DSD in the presence of associated malformations or other system involvement. CNVs may be present in one-fifth of DSD cases, although they are more prevalent in syndromic forms (22, 23, 24). Indeed, several genes regulating sex development have been shown to exert a dose-dependent effect such as duplications of *FGF9*, *SOX3* or *SOX9* in 46,XX DSD and of *DAX1* or *WNT4* in 46,XY DSD as well as deletions of *ATRX*, *DMRT1*, *EMX2* or *WT1* in 46,XY DSD (Table 1). Alterations in gene dosage may be detected by multiplex ligation-dependent probe amplification (MLPA), SNP array or aCGH: MLPA is designed to detect intragenic and whole gene CNV in specific target sites while aCGH is able to detect CNV along the genome. In general SNP array can detect smaller regions of CNV than other more dated platforms, such as oligo-aCGH. These techniques may detect intragenic deletions that may not always be detected by Sanger sequencing of genes (22, 25, 26, 27). CNVs have been reported to be present in one-third of children with DSD (21) and, although in this report three quarters were classified as being of uncertain clinical significance, upon review, half of these had in fact been reported in association with DSD (28). This highlights the importance of diagnostic services being adequately resourced to interpret genetic findings as well as the need for collaboration and data sharing to facilitate the interpretation of these often challenging results (28).

In addition, WES has the potential to detect CNVs in the coding regions of genes at an exon-level resolution, and this is not always feasible when using aCGH or MLPA. In the near future, WGS will be able to detect CNVs in non-coding regulatory regions (29).

### Sanger sequencing

Sanger sequencing of exonic coding, flanking regions and sometimes of other regions of interest (i.e. promoter regions) of a candidate gene could be performed in:

- Targeted (hypothesis-driven) sequencing of known DSD genes following meaningful clinical and hormonal assessment (e.g., *CYP21A2*, *CYP11B1*, *SRD5A2*, *HSD17B3*, *AR* and others) (30).
- Targeted (hypothesis-driven) sequencing of known DSD genes in which HTS methods are currently difficult to interpret due to complex gene rearrangements, deletions, etc. (e.g., *CYP21A2*).
- Sanger sequencing to segregate a specific gene variant in a family.
- Validation of newly set up HTS methods in diagnostic laboratories (DSD panels, WES, WGS) as part of laboratory quality management.
- Validation of HTS data in single cases and their relatives, if necessary based on laboratory quality management.

Individual gene sequencing is increasingly being replaced by HTS of candidate genes or exome, even when clinical and biochemical phenotypes point to a specific gene and based on local and national provision of diagnostic pathways.

### HTS techniques

Due to the highly variable aetiology of DSD, a large number of genes can be considered causative. Moreover, the clinical appearance and the hormonal patterns are often variable, hampering classical hypothesis-driven prediction of a likely causative gene in DSD. Therefore, HTS-based strategies are increasingly replacing traditional sequencing methods, which are laborious and can only analyse one gene at the time. A panel of candidate genes with a robust coverage of all genomic regions of interest is increasingly regarded as the first-tier approach. However, the authors of this COST Action remind the readership that, with the only exception of certain disruptive mutations in some (but not all) DSD-related genes (e.g., the androgen receptor gene and a few more), molecular diagnosis cannot reliably predict the functional consequences for an individual DSD case (e.g., the potential of steroid biosynthesis by the gonads or by the adrenals or the sensitivity to sex steroids), which are important aspects for individualised clinical management.

HTS technologies are continuously improving and the pure technical costs per base are significantly decreasing. However, this does not take into account the increased need for bioinformatic expertise. The specialist resources that are required for multidisciplinary interpretation, explanation and counselling of data following exome or genome sequencing will also need to be taken into account when considering overall costs. The strategy for selecting the best HTS approach will depend on the number of genes on a selected panel, gene coverage and local availability. Strategies for the stepwise molecular investigation of a DSD have been described (8, 31). It is likely that these HTS strategies will further evolve in the near future, based on continuous technical advances. WES-based HTS strategies are more flexible than panel-based HTS strategies and allow the identification of new DSD-related genes. However, the service laboratories and clinicians need to be aware of problems associated when detecting variants of unknown significance (VUS). Increasing experience with HTS techniques suggests that in suspected 46,XY DSD, the identification of CNVs and single gene variants does not always correlate with endocrine profiling (28). Altogether, this may change the classical sequential diagnostic pathway in DSD, favouring a more parallel approach involving integration of both biochemical (hormonal) and genetic (HTS-related) testing, starting with the initial evaluation of the patient (5, 32). As stated earlier, genetic testing and hormonal diagnosis must be considered complementary in DSD. To allow better understanding of DSDs and, hence, improved diagnostic algorithm in the future, this COST Action strongly recommends that molecular data together with clinical and biochemical data in DSDs should be prospectively collected in international databases like I-DSD Registry (33). The I-DSD Registry (www.i-dsd.org) currently has a facility to act as a secure repository for detailed genetic data. Alternatively, this registry can also act as a facility that can signpost investigators to data held in other local repositories.

The experience of different authors reporting results obtained with candidate gene panels designed to analyse a variable number of genes associated with DSD (both 46,XY and 46,XX and even with hypogonadotropic hypogonadism) varies according to panel content and selected patient series (34, 35, 36, 37, 38). An overview of all genes included in at least one of recently reported large DSD gene panels is given in Cools et al (19).

A WES first-line approach would require a much more thorough bioinformatic analysis and may result in a lower guarantee of coverage, thus increasing the possibility of missing variants in a DSD candidate gene. However, many clinical services will probably increase its use as a first-line approach, provided that prices progressively fall, quality further improves, technical hurdles be addressed appropriately and bioinformatic analysis speed up, thus facilitating the broadest view on molecular genetic variations found in DSD.

WES has afforded discovery of genes previously not associated with DSD in humans such as *FOG2/ZFPM2* (39), *HHAT* (40), *FGFR1* (41), *SOX8* (42), *NR2F2* (43) and *ZNRF3* (44) and variants in known genes in previously undescribed phenotypes such as for *NR5A1* (1, 45, 46) or in previously unsuspected genes (47, 48, 49).

When AIS is highly suspected but *AR* gene exon coding and flanking region sequences are normal, whole *AR* gene and promoter regions HTS may reveal pathogenic variants (50). In addition, variants in other genes regulating the *AR* pathway may be suspected in the recently proposed AIS type II patients when *ApoD* expression is not upregulated by androgens in the patient genital skin fibroblasts (51) and will have to be characterised on a molecular level by WES or WGS.

Many of the genetic variants in DSD encode transcription factors such as SRY, SOX9, NR5A1, and FOXL2 and it can be hypothesised that, at least part of the missing genetic variation in severe forms of DSD, can be explained by non-coding variants in regulatory elements (52) that could be detected by WGS. As stated earlier, WES has the potential to detect CNVs in the coding regions while WGS will be able to detect CNVs in non-coding regulatory regions (29). In addition, WES and WGS, will most likely demonstrate that the phenotype of some individuals with DSD result from a combination of gene variations (oligogenic) (15, 16), although monogenic causes will remain for a specific set of phenotypes (i.e. steroid enzyme deficiencies and hormone resistance syndromes such as complete AIS and Leydig cell aplasia/hypoplasia).

### Existing diagnostic algorithms

A number of algorithms have been published to guide the genetic diagnosis of DSD, depending on available clinical data, chromosomal sex, initial hormonal evaluation, presence of associated malformations, functioning testes or Müllerian structures, family history of DSD or reproductive problems and preferred or available local genomic approach. The most general approaches are presented by Croft *et al*. (7) and Alhomaidah *et al*. (5). Their paradigms begin with a DSD phenotype considering the physical features, hormone findings and confirmation of sex chromosome complement, followed by either a targeted gene panel approach or a broad approach involving WES or WGS, the genomic outcomes of each pathway and the consequences for the patients. More detailed algorithms determine suitability for extensive testing through WES or WGS (31) by considering the presence of associated malformations, the results of a-CGH and sex chromosomes, the presence of functioning testes or Müllerian structures, the gene panel results and the presence of a family history. A practical approach in infants with atypical genitalia considers the presence of palpable gonads, the anatomy of the external genitalia, the presence of uterus and the karyotype which guide further chromosomal analyses, hormonal evaluation and genomic testing (9). The most detailed pathway for investigating 46,XY DSD cases without sex chromosome rearrangement requires a multidisciplinary team diagnostic assessment (clinical endocrinology, biochemistry, clinical genetics and molecular genetics), the results of a XY DSD candidate gene panel or of a WES/WGS approach and the need for second-line endocrine tests and functional analyses (*in silico* and/or *in vitro*) to facilitate the interpretation of VUS (5).

The majority of accredited laboratories follow the American College of Medical Genetics and Genomics (ACMG) guidelines for the interpretation of sequence variants and use specific standard terminology (such as ‘pathogenic’, ‘likely pathogenic’, ‘uncertain significance’, ‘likely benign’ and ‘benign’) to describe variants identified in genes that cause Mendelian disorders (53). At present, the general recommendation is to report variants classified as ‘pathogenic’, ‘likely pathogenic’ and of ‘uncertain significance’ in gene(s) related to the patient phenotype, while variants detected by clinical exome and genome sequencing in unrelated genes will be reported as secondary findings (54, 55). With generation of more robust genomic data in this field, these guidelines will evolve as the relationship between the genotype and the phenotype becomes increasingly coherent.

## Recommendations for the genetic molecular diagnosis of DSD

The karyotype will first determine the subgroup of DSD, either chromosomal sex DSD, 46,XX or 46,XY.

A CNV analysis should be added to the first-line diagnostics in both 46,XX and 46,XY, especially if the phenotype includes malformations in other systems in addition to those involved in urogenital development.

All neonates and young babies presenting with DSD need comprehensive steroid analysis in order to avoid missing a potentially life-threatening acute adrenal insufficiency (e.g. in 46,XY DSD due to StAR or P450scc deficiencies, as well as in 46,XX DSD and a virilising form of CAH such as 21-hydroxylase, 11ß-hydroxylase or 3ß-hydroxysteroid dehydrogenase deficiencies) (20). A specific steroid profile in urine and/or plasma may rapidly lead to a likely DSD target gene that can be sequenced on a hypothesis-driven basis by Sanger sequencing or by HTS.

In all other cases, the multidisciplinary team should conduct the physical phenotyping and biochemical (hormonal) evaluation in parallel with the genetic study (Fig. 1). Except for confirming a monogenic familial DSD cause, a HTS panel of candidate genes or WES should be used preferably to analyse candidate genes. WGS is currently reserved for the characterisation of novel DSD genes or in cases with a suspected oligogenic/polygenic basis of the DSD.

These recommendations are based on currently available knowledge and technologies and are to be considered by the professionals and the Health System Organisations on a country-by-country basis. The European Union has recently launched the coordination of reference centres for rare endocrine diseases (Endo-ERN), with the aim of contributing to the availability of adequate patient management. Each country or region should organise the availability of genetic diagnoses following recommendations by multidisciplinary teams for each group of rare diseases. It is evident that both the professionals and the affected persons suffer inequalities: many world countries or regions lack resources to perform even the most basic analyses; even in countries with sufficient resources, a variable percentage of persons lack access to medical attention.

Genetic information and molecular data, together with phenotypic, biochemical and outcome data (when available) in DSDs, should be accessible through international databases like the I-DSD and I-CAH Registries. Databases are important for improving patient care and expanding knowledge about the process of sex determination and development.

## Declaration of interest

The authors declare that there is no conflict of interest that could be perceived as prejudicing the impartiality of this review.

## Funding

This article is based upon work from COST Action BM1303 DSDnet, supported by COST (European Cooperation in Science and Technology). O H (chair of COST Action and chair of working group 5), L A (co-chair of COST Action and working group 3), S A W (chair of working group 3), S F A (chair of working group 4), M C (chair of working group 1), N K (working group 3), K McE (chair of working group 2), P M H (working group 3), A G (working group 2), A B (working group 2) as well as R McG (international partner) appreciate support from BMBS COST Action BM1303.

## Author contribution statement

All the authors have accepted responsibility for the entire content of this submitted manuscript and approved submission.
